# Common genetic variants in pre-microRNAs are associated with cervical cancer susceptibility in southern Chinese women

**DOI:** 10.7150/jca.39636

**Published:** 2020-02-03

**Authors:** Guange Chen, Mingyao Zhang, Jiawei Zhu, Feng Chen, Danyang Yu, Anqi Zhang, Jing He, Wenfeng Hua, Ping Duan

**Affiliations:** 1 Department of Obstetrics and Gynecology, Second Affiliated Hospital and Yuying Children's Hospital of Wenzhou Medical University, Wenzhou 325027, Zhejiang, China; 2 Department of Pediatric Surgery, Guangzhou Institute of Pediatrics, Guangzhou Women and Children's Medical Center, Guangzhou Medical University, Guangzhou 510623, Guangdong, China; 3 Department of Laboratory Medicine and Central Laboratories, Guangdong Second Provincial General Hospital, Guangzhou 510317, Guangdong, China

**Keywords:** case-control study, cervical cancer, pre-microRNA, polymorphism, genetic susceptibility

## Abstract

Cervical cancer is a commonly diagnosed cancer among females. Polymorphisms in pre-microRNAs have been demonstrated to play critical roles in cancer. However, the roles of pre-microRNA polymorphisms in the aetiology of cervical cancer have not been well documented. We genotyped eight pre-microRNA polymorphisms in 290 cervical cancer patients and 445 cancer-free female controls using quantitative polymerase chain reaction with TaqMan probes. To estimate the association between pre-microRNA polymorphisms and the risk of cervical cancer, an unconditional logistic regression model was used to calculate the odds ratio (OR) and 95% confidence interval (CI), adjusting for age, menopause, delivery, and abortion. We found that the *pre-miR-137* rs1625579 T > G polymorphism was associated with a significant decrease in cervical cancer risk (TG/GG versus TT: adjusted OR (AOR) = 0.47, 95% CI = 0.27-0.81; TG versus TT: AOR = 0.56, 95% CI = 0.34-0.91). We also observed a significant association between the *pre-miR-27a* rs895819 T > C polymorphism and decreased cervical cancer risk (TC/CC versus TT: AOR = 0.65, 95% CI = 0.44-0.96). Stratified analysis further demonstrated that the *pre-miR-137* rs1625579 T > C and *pre-miR-27a* rs895819 T > C polymorphisms significantly reduced the risk of cervical cancer susceptibility in patients younger than 49 years, those who experienced fewer abortions, and clinical stage I patients. Moreover, the *pre-miR-137* rs1625579 T > G polymorphism showed protective effects in premenopausal women, squamous cell carcinoma patients, and patients with unclassified types of pathologies; the *pre-miR-27a* rs895819 T > C polymorphism was also associated with a decreased risk in patients older than 49 years, menopausal women, and women who had experienced vaginal pregnancies. The *pre-miR-137* rs1625579 T > G and *pre-miR-27a* rs895819 T > C polymorphisms may provide protective effects against susceptibility to cervical cancer risk.

## Introduction

Cervical cancer was the fourth leading cause of cancer death and the fourth most frequently diagnosed cancer among females worldwide in 2018 1. In China, 26,400 and 30,500 estimated deaths from cervical cancer were reported in 2013 and 2015, respectively 2, 3. High-risk strains of human papillomavirus (HPV) are now well established as the causative agents responsible for invasive cervical cancer and its precursor, cervical intraepithelial neoplasia 4. HPV infection is strongly associated with the risk of cervical cancer. Only a small number of women exhibit persistent infection with oncogenic HPV types that eventually lead to cervical cancer, and most HPV infections regress spontaneously 5. Increasing evidence indicates that host genetic variations play critical roles in cervical carcinogenesis independent of HPV infection 6-9.

MicroRNAs (miRNAs) are a class of small regulatory RNAs that are processed from endogenous hairpin transcripts and function as regulators of gene expression in eukaryotes 10, 11. In the nucleus, miRNAs are transcribed from genomic DNA into long primary transcripts (pri-miRNAs) and can be cleaved into 60-70-nucleotide precursor miRNAs (pre-miRNAs) by nuclear Drosha 12, 13. Next, the pre-miRNAs are further processed into mature miRNAs of approximately 21-25 nucleotides in length 14, 15. Polymorphisms or mutations in the promoter or the miRNA sequence itself may result in changes in the structure or expression of miRNAs, which affect the expression of hundreds of target genes. Cancer susceptibility and prognosis cancer may be affected by polymorphisms in miRNAs 16. Recent studies have shown that the genetic variants in pre-miRNAs are associated with the risks and clinical outcomes of many types of cancers 17-20, including cervical cancer 7, 21. In this study, we sought to identify common genetic polymorphisms of pre-miRNAs associated with the risk of cervical cancer susceptibility in southern Chinese women.

## Materials and Methods

### Patients and controls

In the present case-control study, 290 cervical cancer patients (mean ± standard deviation (SD), 50.10 ± 10.51) were enrolled at the Second Affiliated Hospital and Yuying Children's Hospital of Wenzhou Medical University (WMU) from February 2007 to February 2017. The diagnosis of cervical cancer was confirmed in all cases by histological examination of tissues from biopsies or resected specimens. Cancer-free controls (n=535, mean ± SD, 57.84 ± 15.47) were recruited from the same hospital during routine physical examinations, and we excluded participants who had been diagnosed with malignant neoplasm or a family history of cancers. This study was approved by the Second Affiliated Hospital and Yuying Children's Hospital of WMU (Ethics Reference No: LCKY2019-274), and written informed consent was obtained from all patients.

### Polymorphism selection and genotyping

Eight widely investigated polymorphisms (*pre-miR-137* rs1625579 T>G, *pre-miR-27a* rs895819 T>C, *pre-miR-146a* rs2910164 C > G, *pre-miR-149* rs2292832 T>C, *pre-miR-196a2* rs11614913 T>C, *pre-miR-218* rs11134527 A>G, *pre-miR-423* rs6505162 C>A, and *pre-miR-608* rs4919510 G>C) were selected 17. The minor allele frequency of all eight polymorphisms was greater than 0.05. Seven of the polymorphisms were located in transcription factor binding sites, as predicted by SNPinfo (https://snpinfo.niehs.nih.gov/), and the rs1625579 polymorphism is located in an intron of the *miR-137* gene, which is significantly associated with schizophrenia risk 22.

The TIAN quick FFPE DNA Kit (Qiagen NV, Venlo, the Netherlands) was applied to extract genomic DNA from all patients from paraffin-embedded tissues, while genomic DNA from the controls was extracted from peripheral blood specimens using the TIANamp Blood DNA Kit (TianGenBiotech, Beijing, China) as we described previously 23, 24. A UV absorption spectrophotometer was used to detect DNA purity and concentrations.

Genotyping analysis was performed by real-time PCR with TaqMan PCR master mix and the ABI Prism 7900HT genetic detection system. The details of the analysis procedures have been previously described 25. In addition, approximately 5% of the samples were randomly selected as positive and negative controls for assessing the accuracy of genotyping results.

### Statistical analysis

The goodness-of-fit chi-square test was adopted to evaluate departure from Hardy-Weinberg equilibrium (HWE) for the selected polymorphisms in control subjects. The heterogeneity of the genotypes and ages between patients and controls was evaluated by using a 2-sided chi-square test. The association between pre-miRNA polymorphisms and cervical cancer risk was assessed with an unconditional logistic regression model, calculated as the crude and adjusted odds ratios (OR) and 95% confidence intervals (95% CI). Additionally, stratified analyses were performed by age, menopause, the number of vaginal pregnancies, the number of abortions, clinical stages and pathological stages. All statistical tests were carried out by using SAS software (Version 9.4; SAS Institute, Cary, NC, USA), with a two-sided *P*-value < 0.05 considering significant controls.

## Results

### Characteristics of the study participants

In the present study, we enrolled 290 cervical cancer patients and 445 cancer-free controls. The frequency distributions of the selected demographic characteristics of the cases and controls are shown in Table 1. The incidence of abortion in the patients (43.5%) was much higher than that in the control subjects (4.9%), implying that a high incidence of abortion was a critical risk factor for cervical cancer development in our study population. In addition, compared with the controls, the distribution of age in the cervical cancer cases was much younger. Among the 290 cases, 105 (36.2%) were postmenopausal women. However, there was no significant difference in the number of vaginal deliveries between the cases and controls. There were 208 patients (71.7%) who had squamous cell carcinomas; other patients who had adenocarcinoma or adenosquamous carcinoma or were unclassified were represented 28.3% of the study population. Among all 290 cases, 70.0% of patients were classified as stage I and 21.0%, 1.7%, and 0.7% as stages II, III, and IV, respectively, while 6.6% of the patients were classified as being in other stages (Table 1).

### Association between selected pre-miRNA polymorphisms and cervical cancer risk

To investigate the association with cervical cancer risk of each of the eight selected pre-miRNA polymorphisms, the HWE test was first used to identify genotyping errors in the cancer-free controls. As shown in Table 2, most of the genotype distributions were in accordance with HWE (*P* values ranged from 0.215 to 0.953), except that the pre-miR-149 rs2292832 T > C and pre-miR-218 rs11134527 A > G polymorphisms exhibited significant deviation from HWE in the controls (*P*<0.001). Next, through allelic association analysis, we found that the pre-miR-137 rs1625579 T > G polymorphism was significantly associated with decreased cervical cancer risk (TG/GG versus TT: adjusted odds ratio (AOR) = 0.47, 95% CI = 0.27-0.81, *P*=0.007; TG versus TT: AOR = 0.56, 95% CI = 0.34-0.91, *P*=0.018). The pre-miR-27a rs895819 T > C polymorphism was also shown to significantly decrease cervical cancer susceptibility (TC/CC versus TT: AOR = 0.65, 95% CI = 0.44-0.96, *P*=0.030). These findings suggest that these two polymorphisms may be common pre-miRNA polymorphisms that influence the risk of cervical cancer susceptibility in southern Chinese women.

### Stratified analysis

We further explored the association of the pre-miR-137 rs1625579 T > G and pre-miR-27a rs895819 T > C polymorphisms with cervical cancer susceptibility through stratified analysis. We found that the protective effects of the pre-miR-137 rs1625579 T > G and pre-miR-27a rs895819 T > C polymorphisms were significant in patients younger than 49 years, those exhibiting fewer abortions, and those in clinical stage I (Table 3). Moreover, the protective effect of the *pre-miR-137* rs1625579 T > G polymorphism was also observed in premenopausal women, patients with squamous cell carcinoma, and patients with unclassified types of pathologies, while the *pre-miR-27a* rs895819 T > C polymorphism was also associated a low risk of cervical cancer susceptibility in patients older than 49 years, those who were menopausal, and those who had experienced vaginal pregnancies (Table 3).

## Discussion

In the current case-control study, we explored the associations of eight common polymorphisms in pre-miRNAs with cervical cancer susceptibility in southern Chinese women. We found that the *pre-miR-137* rs1625579 T>G and *pre-miR-27a* rs895819 T > C polymorphisms were significantly associated with decreased cervical cancer risk. Our findings suggest that the *pre-miR-137* rs1625579 T>G and *pre-miR-27a* rs895819 T > C polymorphisms may play critical roles in the aetiology of cervical cancer.

Tumour-suppressive functions of miR-137 in cervical cancer have been recently reported 26. Endogenous miR-137 expression is downregulated in both cervical cancer cell lines and tissues, and overexpression of miR-137 in cervical cancer cells substantially inhibits cell proliferation, migration and transplantation in nude mice 26. In our study, the results showed that the *pre-miR-137* rs1625579 T>G polymorphism exhibits a protective effect against cervical cancer, suggesting that the *pre-miR-137* rs1625579 T>G polymorphism may present a positive correlation with miR-137 expression. Further studies are needed to confirm this hypothesis.

Remarkably, we also found that the *pre-miR-27a* rs895819 T>C polymorphism exerted a protective effect on cervical cancer risk. This finding is consistent with a previous study conducted in cervical cancer 7. It is worth noting that our results demonstrated that the *pre-miR-27a* rs895819 T>C polymorphism exerted a protective effect against cervical cancer risk in dominant genetic model, while Xiong et al. found that the *pre-miR-27a* rs895819 T>C polymorphism played a protective role in a recessive genetic model but found no associations in other genetic models 7. Although the participants of Xiong et al.' study and our study were both selected from among southern Chinese women, the sample size in the current study was larger than the sample size in the Xiong et al. study (including 103 cervical cancer patients and 417 cancer-free controls). Thus, the differences may result from the baseline characteristics and the larger number of samples.

Additionally, previous studies have suggested that the *pre-miR-149* rs2292832 T > C polymorphism is associated with an increased risk of cervical cancer susceptibility 27 and that the *pre-miR-218* rs11134527 A > G polymorphism is associated with a decreased risk of cervical cancer susceptibility 28, 29. However, our study did not identify a significant association of these two polymorphisms with cervical cancer risk. This inconsistency with previous studies may be due to the genotype distributions of the *pre-miR-149* rs2292832 T > C and *pre-miR-218* rs11134527 A > G polymorphisms significantly deviating from HWE in the controls. For the other pre-miRNA polymorphisms, no associations were found with the risk of cervical cancer susceptibility in the current study, so further studies are required for confirmation.

Although the current case-control study provides evidence that common pre-miRNA polymorphisms are associated with the risk of cervical cancer susceptibility in southern Chinese women, several limitations should be addressed. First, only eight common pre-miRNA polymorphisms were genotyped in the current study; hence, more common pre-miRNA polymorphisms should be investigated to fully illuminate the contribution of polymorphisms in pre-miRNAs to cervical cancer susceptibility. Second, the sample size in the current study was relatively small. Additionally, the selected samples came from a single hospital in the current hospital-based case-control study, which may result in selection bias or even a decreased or increased risk assessment. Third, in addition to polymorphisms, many that confounders may also influence susceptibility to cervical cancer, such as gene-gene interactions, gene-environment interactions, and the specific tumour pathologic classification, were not taken into consideration due to the lack of individual information. Fourth, although our findings suggested that the *pre-miR-137* rs1625579 T > G and *pre-miR-27a* rs895819 T > C polymorphisms show a statistically significant association with the risk of cervical cancer, much more research is needed to confirm this result and to detect the potential related mechanisms and functions in the future.

## Supplementary Material

Supplementary tables.

## Figures and Tables

**Table 1:** Frequency distribution of selected variables in cervical cancer cases and cancer-free controls

Characteristic	Cases (n=290)	Controls (n=445)	*P^a^*
	No. (%)	No. (%)	
**Age**			
Mean ± SD	50.10 ± 10.51	57.84 ± 15.47	
≤49	150 (51.7%)	118 (26.5%)	<0.0001
>49	140 (48.28%)	327 (73.5%)	
**Menopause**			
No	185 (63.8%)	131 (29.4%)	<0.0001
Yes	105 (36.2%)	314 (70.6%)	
**Delivery**			
No	2 (0.71%)	10 (2.30%)	0.107
Yes	279 (99.3%)	425 (97.7%)	
**Abortion**			
No	164 (56.6%)	423 (95.1%)	<0.0001
Yes	126 (43.5%)	22 (4.9%)	
**Pathology**			
Squamous cell carcinoma	208 (71.7%)		
Adenocarcinoma	44 (15.2%)		
Adenosquamous carcinoma	3 (1.0%)		
Other	35 (12.1%)		
**FIGO stages**			
I	203 (70.0%)		
II	61 (21.0%)		
III	5 (1.7%)		
IV	2 (0.7%)		
Others	19 (6.6%)		

**^a^**Two-sided χ^2^ test for the distribution between cervical cancer cases and cancer-free controls.

**Table 2:** Association between selected polymorphisms and cervical cancer determined by logistic regression analyses

miRNA	SNP	Allele		Case (N=290)			Control (N=445)			Dominant		Recessive		Heterozygous		HWE
		A	B	AA	AB	BB	AA	AB	BB	AOR (95% CI)	*P ^a^*	AOR (95% CI)	*P* ^a^	AOR (95% CI)	*P* ^a^	
*miR-137*	rs1625579	T	G	254	26	7	361	75	5	0.47 (0.27-0.81)	0.007	1.19 (0.23-6.25)	0.834	0.56 (0.34-0.91)	0.018	0.621
*miR-27a*	rs895819	T	C	202	76	11	252	158	31	0.65 (0.44-0.96)	0.030	1.01 (0.46-2.23)	0.980	0.75 (0.55-1.04)	0.083	0.365
*miR-146a*	rs2910164	C	G	118	123	49	152	209	80	0.74 (0.51-1.09)	0.129	0.84 (0.52-1.36)	0.479	0.83 (0.64-1.08)	0.162	0.582
*miR-149*	rs2292832	T	C	185	57	28	309	76	42	1.07 (0.71-1.64)	0.741	0.94 (0.50-1.79)	0.859	1.02 (0.77-1.36)	0.885	<0.001
*miR-196a2*	rs11614913	T	C	105	125	58	140	220	80	0.72 (0.49-1.07)	0.100	1.26 (0.79-2.00)	0.330	0.93 (0.72-1.21)	0.585	0.691
*miR-218*	rs11134527	A	G	93	123	61	185	160	85	1.16 (0.79-1.71)	0.460	1.22 (0.77-1.95)	0.400	1.13 (0.88-1.45)	0.350	<0.001
*miR-423*	rs6505162	C	A	180	89	11	291	120	18	1.48 (1.01-2.19)	0.057	1.86 (0.81-4.24)	0.142	1.43 (1.04-1.96)	0.029	0.215
*miR-608*	rs4919510	G	C	108	129	51	125	219	97	0.74 (0.50-1.11)	0.144	1.02 (0.65-1.61)	0.928	0.89 (0.69-1.15)	0.372	0.953

OR, odds ratio; CI, confidence interval. HWE, Hardy-Weinberg equilibrium. Heterozygous (AB versus AA), dominant (AB/BB versus AA), recessive (BB versus AB/AA). ^a^Adjusted for age, menopause, number of vaginal pregnancies, abortion, clinical stages and pathology.

**Table 3:** Stratification analysis for the associations of the rs895819 T > C and rs1625579 T > G polymorphisms with cervical cancer risk

Variables	*miR-137* rs1625579 (cases/controls)		AOR (95% CI)	*P*^a^	*miR-27a* rs895819 (cases/controls)		AOR (95% CI)	*P*^a^
	TT	TG/GG			TT	TC/CC		
**Age (years)**								
≤49	133/89	15/28	0.36 (0.18-0.71)	0.003	106/68	44/50	0.57 (0.34-0.94)	0.027
>49	121/272	18/52	0.78 (0.44-1.39)	0.394	196/184	43/139	0.59 (0.39-0.90)	0.015
**Menopause**								
No	161/99	22/31	0.44 (0.24-0.80)	0.007	124/78	60/53	0.71 (0.45-1.13)	0.153
Yes	93/262	11/49	0.63 (0.32-1.27)	0.197	78/174	27/136	0.44 (0.27-0.72)	0.001
**Delivery**								
No	1/9	1/1	9.00 (0.28-285.45)	0.213	2/7	0/3	-	0.962
Yes	245/346	31/75	0.58 (0.37-0.92)	0.019	195/242	83/179	0.58 (0.42-0.79)	0.008
**Abortion**								
No	146/350	16/78	0.49 (0.28-0.87)	0.015	110/243	54/185	0.65 (0.44-0.94)	0.023
Yes	108/11	17/2	0.87 (0.18-4.25)	0.859	92/9	33/4	0.81 (0.23-2.80)	0.735
**Clinical stage**								
I	177/361	24/80	0.45 (0.24-0.82)	0.010	142/252	60/189	0.64 (0.42-0.98)	0.041
II	53/361	7/80	0.55 (0.23-1.34)	0.190	44/252	17/189	0.61 (0.32-1.16)	0.133
III	4/361	1/80	1.47 (0.14-15.34)	0.746	3/252	2/189	1.19 (0.16-8.68)	0.868
IV	2/361	0/80	-	-	1/252	1/189	-	-
Others	18/361	1/80	0.29 (0.04-2.34)	0.246	12/252	7/189	0.48 (0.14-1.63)	0.241
**Pathology**								
Squamous cell carcinoma	180/361	25/80	0.51 (0.29-0.92)	0.024	143/252	64/189	0.68 (0.45-1.02)	0.065
Adenocarcinoma	38/361	6/80	0.65 (0.24-1.72)	0.380	32/252	12/189	0.52 (0.24-1.14)	0.102
Adenosquamous carcinoma	3/361	0/80	-	-	2/252	1/189	-	-
Others	33/361	2/80	0.16 (0.03-0.87)	0.034	25/252	10/189	0.54 (0.22-1.32)	0.177

AOR, adjusted odds ratio; CI, confidence interval. ^a^ Obtained in logistic regression models with adjustment for age, menopause, delivery, and abortion.

## Abbreviations

HPV: human papillomavirus
miRNAs: microRNAs
pre-miRNAs: precursor miRNAs
HWE: Hardy-Weinberg equilibrium
OR: odds ratio
CI: confidence interval
AOR: adjusted odds ratios

## References

[1] Bray F, Ferlay J, Soerjomataram I, Siegel RL, Torre LA, Jemal A (2018). Global cancer statistics 2018: GLOBOCAN estimates of incidence and mortality worldwide for 36 cancers in 185 countries. CA: a cancer journal for clinicians.

[2] Xia C, Ding C, Zheng R, Zhang S, Zeng H, Wang J (2017). Trends in geographical disparities for cervical cancer mortality in China from 1973 to 2013: a subnational spatio-temporal study. Chin J Cancer Res.

[3] Chen W, Zheng R, Baade PD, Zhang S, Zeng H, Bray F (2016). Cancer statistics in China, 2015. CA: a cancer journal for clinicians.

[4] Bosch FX, Lorincz A, Munoz N, Meijer CJ, Shah KV (2002). The causal relation between human papillomavirus and cervical cancer. Journal of clinical pathology.

[5] Dunne EF, Park IU (2013). HPV and HPV-associated diseases. Infectious disease clinics of North America.

[6] de Freitas AC, Gurgel AP, Chagas BS, Coimbra EC, do Amaral CM (2012). Susceptibility to cervical cancer: an overview. Gynecol Oncol.

[7] Xiong XD, Luo XP, Cheng J, Liu X, Li EM, Zeng LQ (2014). A genetic variant in pre-miR-27a is associated with a reduced cervical cancer risk in southern Chinese women. Gynecologic oncology.

[8] Johanneson B, Chen D, Enroth S, Cui T, Gyllensten U (2014). Systematic validation of hypothesis-driven candidate genes for cervical cancer in a genome-wide association study. Carcinogenesis.

[9] Chen D, Gyllensten U (2015). Lessons and implications from association studies and post-GWAS analyses of cervical cancer. Trends in genetics: TIG.

[10] Bentwich I, Avniel A, Karov Y, Aharonov R, Gilad S, Barad O (2005). Identification of hundreds of conserved and nonconserved human microRNAs. Nat Genet.

[11] Bartel DP (2018). Metazoan MicroRNAs. Cell.

[12] Schanen BC, Li X (2011). Transcriptional regulation of mammalian miRNA genes. Genomics.

[13] Yoontae Lee1 KJ, Jun-Tae Lee1, Sunyoung Kim1, 2 and, V.Narry Kim2 (2002). MicroRNA maturation: stepwise processing and subcellular localization. The EMBO Journal.

[14] Lund E, Guttinger S, Calado A, Dahlberg JE, Kutay U (2004). Nuclear export of microRNA precursors. Science.

[15] Hutvágner G, Mclachlan J, Pasquinelli AE, Bálint E, Tuschl T, Zamore PDJS (2001). A cellular function for the RNA-interference enzyme Dicer in the maturation of the let-7 small temporal RNA. Science.

[16] Ryan BM, Robles AI, Harris CC (2010). Genetic variation in microRNA networks: the implications for cancer research. Nature reviews Cancer.

[17] He J, Zou Y, Liu X, Zhu J, Zhang J, Zhang R (2018). Association of Common Genetic Variants in Pre-microRNAs and Neuroblastoma Susceptibility: A Two-Center Study in Chinese Children. Molecular therapy Nucleic acids.

[18] Martin-Guerrero I, Gutierrez-Camino A, Lopez-Lopez E, Bilbao-Aldaiturriaga N, Pombar-Gomez M, Ardanaz M (2015). Genetic variants in miRNA processing genes and pre-miRNAs are associated with the risk of chronic lymphocytic leukemia. PloS one.

[19] Bensen JT, Tse CK, Nyante SJ, Barnholtz-Sloan JS, Cole SR, Millikan RC (2013). Association of germline microRNA SNPs in pre-miRNA flanking region and breast cancer risk and survival: the Carolina Breast Cancer Study. Cancer Causes Control.

[20] Yin Z, Cui Z, Ren Y, Xia L, Wang Q, Zhang Y (2016). Association between polymorphisms in pre-miRNA genes and risk of lung cancer in a Chinese non-smoking female population. Lung cancer.

[21] Zhou B, Wang K, Wang Y, Xi M, Zhang Z, Song Y (2011). Common genetic polymorphisms in pre-microRNAs and risk of cervical squamous cell carcinoma. Molecular carcinogenesis.

[22] Schizophrenia Psychiatric Genome-Wide Association Study C (2011). Genome-wide association study identifies five new schizophrenia loci. Nature genetics.

[23] He J, Wang F, Zhu J, Zhang R, Yang T, Zou Y (2016). Association of potentially functional variants in the XPG gene with neuroblastoma risk in a Chinese population. Journal of cellular and molecular medicine.

[24] He J, Wang F, Zhu J, Zhang Z, Zou Y, Zhang R (2017). The TP53 gene rs1042522 C>G polymorphism and neuroblastoma risk in Chinese children. Aging.

[25] He J, Qiu LX, Wang MY, Hua RX, Zhang RX, Yu HP (2012). Polymorphisms in the XPG gene and risk of gastric cancer in Chinese populations. Human genetics.

[26] Zhang H, Yan T, Liu Z, Wang J, Lu Y, Li D (2018). MicroRNA-137 is negatively associated with clinical outcome and regulates tumor development through EZH2 in cervical cancer. Journal of cellular biochemistry.

[27] Wang S, Zhu H, Ding B, Feng X, Zhao W, Cui M (2019). Genetic variants in microRNAs are associated with cervical cancer risk. Mutagenesis.

[28] Zhou X, Chen X, Hu L, Han S, Qiang F, Wu Y (2010). Polymorphisms involved in the miR-218-LAMB3 pathway and susceptibility of cervical cancer, a case-control study in Chinese women. Gynecol Oncol.

[29] Shi TY, Chen XJ, Zhu ML, Wang MY, He J, Yu KD (2013). A pri-miR-218 variant and risk of cervical carcinoma in Chinese women. BMC Cancer.

