# Supplementary material for: Common genetic variants in pre-microRNAs are associated with cervical cancer susceptibility in southern Chinese women
Source: J Cancer. 2020 Feb 3;11(8):2133–8. doi: 10.7150/jca.39636 (PMC7052933; doi:10.7150/jca.39636)
Supplement: Supplementary file 1 — Supplementary tables. [file jcav11p2133s1.pdf]

Supplementary Table S1. Function of selected polymorphisms as predicted by SNPinfo (http://snpinfo.niehs.nih.gov/) software

miRNA	rs	Chr	Allele	TFBS	Splicing (ESE or ESS)	Allele	Asian	CHB
miR-27a	rs895819	19	C/T	Y	Y	T	--	0.711
miR-137	rs1625579	1	T/G	--	--	T	0.947	0.940
miR-146a	rs2910164	5	C/G	Y	Y	G	0.469	0.446
miR-149	rs2292832	2	C/T	Y	Y	C	0.339	0.315
miR-196a2	rs11614913	12	C/T	Y	Y	C	0.427	0.411
miR-218	rs11134527	5	A/G	Y	--	G	0.417	0.367
miR-423	rs6505162	17	A/C	Y	Y	C	0.811	0.833
miR-608	rs4919510	10	C/G	Y	Y	C	0.491	0.488

TFBS, transcription factor binding sites; ESE, exon splicing enhancer; ESS, exon splicing silencer; CHB, Han Chinese in Beijing, China.

Supplementary Table S2. The informed consent and ethics statement.

温州医科大学附属第二医院
育英儿童医院 | 第二临床医学院
The 2nd Affiliated Hospital and Yuying Children's Hospital of WMU / The 2nd School of Medicine, WMU

地址Add:浙江省温州市学院西路109号 邮编Pc.325027
109 Xueyuan west Road Wenzhou Zhejiang PR China
电话Tel.0577-88816381 邮箱E-mail:yb@wzhealth.com
传真Fax:0577-88832693 网址Web:http //www.wzhealth.com/

Date: Aug.16.2019
Applicants: Ping Duan
Department: Department of Obstetrics and Gynecology, the Second Affiliated Hospital of Wenzhou Medical University

**Ethics Reference No:** *Please quote this ref on all correspondence*	LCKY2019-274
**Project Title:**	Common genetic variants in pre-microRNAs are associated with cervical cancer susceptibility in Southern Chinese women
**Researcher' s Name(s):**	Guange Chen, Mingyao Zhang, Jiawei Zhu, Feng Chen, Danyang Yu, Anqi Zhang, Jing He, Wenfeng Hua,*, Ping Duan1*
**Supervisor(s)**	Yue Hu

Thank you for your recent application for ethics approval for the above project. This has now been considered at the Second Affiliated Hospital of Wenzhou Medical University Research Ethics Committee meeting, and I am pleased to inform you that ethics approval has been given to your research.

Please note that, if your research should change significantly in any respect, or if risk of harm or breach of confidentiality becomes likely, you will be obliged to submit a new application.

We wish you every success with your research.

Yours sincerely,

Xue-qiong Zhu Xueqiong Zhu

Research Ethics Committee of the Second Affiliated Hospital of Wenzhou Medical University

诚信 奉献 求精 创新
